# Generative AI: Opportunities, risks, and responsibilities for oral sciences

**DOI:** 10.1016/j.jfscie.2025.100056

**Published:** 2025-10-15

**Authors:** Falk Schwendicke, Sharanbir K. Sidhu, Jack L. Ferracane, Antonin Tichy, Nicholas S. Jakubovics

**Affiliations:** a Clinic for Conservative Dentistry and Periodontology, LMU Clinics, Munich, Germany; b Institute of Dentistry, Barts & The London School of Medicine and Dentistry, Queen Mary University of London, London, UK; c Department of Oral Rehabilitation and Biosciences, Oregon Health & Science University, Portland, OR; d Institute of Dental Medicine, First Faculty of Medicine, Charles University, Prague, Czech Republic; e School of Dental Sciences, Faculty of Medical Sciences, Newcastle University, Framlington Place, Newcastle upon Tyne, UK

**Keywords:** Artificial intelligence, large language models, peer review, reproducibility of results, responsible artificial intelligence, scientific misconduct

## Abstract

Generative artificial intelligence (AI) has the capability to generate new content—including text, code, imagery, video, and speech—based on human prompts and is entering dental and oral research. By retrieving, analyzing, summarizing, and contextualizing vast datasets, generative AI offers substantial potential to enhance scientific workflows. It can improve documentation, communication, and reproducibility while saving time and accelerating discovery. However, its integration into research brings significant ethical, societal, and scientific challenges. Concerns include embedded data biases, automation bias, overreliance, and error propagation, all requiring critical human oversight. Furthermore, generative AI raises complex issues around plagiarism, fraud, attribution, and reproducibility, compounded by the potential for AI “hallucinations” or fabricated content. Addressing these concerns demands transparency, robust verification processes, ethical compliance, and clear documentation distinguishing synthetic from real-world data. Several scientific and regulatory bodies have published guidelines to support responsible AI use. Recommendations relevant to scientists in dental, oral, and craniofacial research include transparent disclosure of AI tools and methods, thorough verification of AI outputs, ethical oversight, and active monitoring. Scientists are urged to work collaboratively with stakeholders to enforce these principles and engage the public in the evolving discourse. The risk of misuse, particularly through fraudulent AI-generated publications, is growing. Paper mills exploiting generative AI can produce fabricated or manipulated articles, which may mislead the scientific community and distort evidence bases. Coordinated action, involving journals, institutions, and ethics bodies, is essential to combat these threats. As generative AI continues to evolve, adaptive and harmonized guidelines wil be necessary to safeguard scientific integrity. Researchers, reviewers, and editors must play a proactive role in ensuring that AI serves to advance—not undermine—the quality and trustworthiness of dental and oral science.

## Perspective

Generative artificial intelligence (AI) is trained on extensive volumes of data and can produce new content including text, code, imagery, video, or speech based on human instructions (also known as prompts). Generative AI also has the potential to significantly enhance research by retrieving, analyzing, summarizing, and contextualizing data; improving documentation, communication, and, hence, reproducibility and transparency; saving time and resources, and overall accelerating research and discovery at higher efficiency. In parallel, generative AI comes with a wealth of ethical, societal, and scientific challenges, among them:

• biases rooted in the learned data (which need identification and proactive mitigation);
• risks of overreliance, automation bias, and error propagation (which can be addressed only by humans understanding and confirming an AI’s output);
• plagiarism, fraud, and attribution problems (which require clarity and disclosure of AI-generated results or analyses but may also be addressed by watermarking of AI-generated texts or AI itself being able to detect such generated content); and
• lacking reproducibility and challenges in verifying an AI’s output, particularly given the risk of “hallucination” of generative AI (which calls for comprehensive and transparent reporting around the usage of AI and scientists developing the skills necessary for critically appraising generative AI products).^1^, ^2^, ^3^

A number of guidelines have been published on the use of generative AI in science.^4^, ^5^, ^6^ The National Academy of Sciences recently convened a panel of experts from academia, industry, and government, leading to a commissioned position paper aiming to address the outlined challenges.^3^ The following key recommendations, which the *Journal of Dental Research* and *JADA Foundational Science* support, have been made toward the usage of generative AI in dental, oral, and craniofacial sciences. We focus here on only those recommendations for scientists, as recommendations for model developers in dentistry have been published in detail elsewhere^7^^,^^8^:

• Transparent disclosure and attribution: Scientists should disclose the use of generative AI and provide details on the tools, algorithms, and settings used, ensuring that readers can attribute human and AI contributions.
• Verification: Scientists using generative AI should proactively use methods to verify AI-generated content, identify possible biases, and provide supportive real-world evidence.
• Documentation: AI-generated (synthetic) data should be clearly marked and distinguished from real-world observations.
• Ethics and equity: Scientists should adhere to guidelines regarding the attribution, intellectual property, privacy, and consent, and address biases in AI systems that may cause harm and increase inequities. Human oversight and accountability remain key in relevant scientific processes, peer-review among them.
• Continuous monitoring and public engagement: Scientists, jointly with other stakeholders in research, industry, government, and civil society, should actively and continuously enforce these principles.

As generative AI becomes embedded in everyday life and scientific workflows, the potential for its misuse must not be underestimated.^9^ Large language models can be exploited to fabricate scientific content, as current safeguards are ineffective in preventing the generation of disinformation.^10^ Entire credible-looking articles can be produced using AI, despite often containing factual or methodological errors.^11^ This makes it increasingly difficult for researchers to distinguish authentic work from fabricated material when reading and reviewing scientific literature.^12^

The misuse of generative AI also involves unauthorized or unacknowledged manipulation of text and results or data fabrication, all of which can occur at any stage of the publishing process. Of particular concern is the emergence of so-called “paper mills,” which use generative AI to produce falsified articles that may be wrongfully attributed to credible authors with no connection to the fraudulent work. Such articles may contain fabricated data, but review articles in which no new data need to be generated are especially likely targets. Addressing fraudulent publications or journals requires coordinated action across the scientific community. Provided that the claims are supported by detailed evidence of the misconduct, the steps may include (1) informing the journal or publisher; (2) checking with established whitelists or blacklists of potential predatory journals and publishers such as those at predatoryjournals.org; (3) notifying relevant authorities, such as the institution(s) with which the authors are affiliated, or regulatory bodies such as ethics committees; and (4) using formal platforms for complaints through organizations such as the Committee on Publication Ethics (COPE).^13^ The consequences of fraudulent publications, including those based on improper usage of generative AI, include misdirection of scientific effort, distortion of evidence syntheses, and potential harm to patient care. In addition, they may cause damage to the reputation of individuals or institutions who are falsely implicated or whose work has been misrepresented.

The chances and risks associated with generative AI for science cannot be fully anticipated at present; guidelines will have to adapt and evolve given the dynamics of the field. No guideline will stand on its own; it will become part of a web of complementary documents that not only foster good scientific practice but also contribute to the emerging regulatory and legislative landscape around AI.^14^ Researchers, peer reviewers, and editors, as well as readers of the journal, are essential in enforcing any guideline and shaping how AI can best serve the dental and oral science.

## Disclosure

The authors declared no potential conflicts of interest with respect to the research, authorship, or publication of this article.
